# Enhanced Recovery After Surgery (ERAS) Protocols in Cardiac Surgery: Impact on Opioid Consumption

**DOI:** 10.3390/jcm14051768

**Published:** 2025-03-06

**Authors:** Alexandra Othenin-Girard, Zied Ltaief, Mario Verdugo-Marchese, Luc Lavanchy, Patrice Vuadens, Anna Nowacka, Ziyad Gunga, Valentine Melly, Tamila Abdurashidova, Caroline Botteau, Marius Hennemann, Jérôme Graf, Patrick Schoettker, Matthias Kirsch, Valentina Rancati

**Affiliations:** 1 Department of Anesthesia, Lausanne University Hospital (CHUV), 1011 Lausanne, Switzerland; alexandra.othenin-girard@chuv.ch (A.O.-G.); luc.lavanchy@chuv.ch (L.L.); patrice.vuadens@chuv.ch (P.V.); patrick.schoettker@chuv.ch (P.S.); 2 Department of Intensive Care, Lausanne University Hospital (CHUV), 1011 Lausanne, Switzerland; zied.ltaief@chuv.ch; 3 Department of Cardiac Surgery, Lausanne University Hospital (CHUV), 1011 Lausanne, Switzerland; mario.verdugo-marchese@chuv.ch (M.V.-M.); anna.nowacka@chuv.ch (A.N.); ziyad.gunga@chuv.ch (Z.G.); valentine.melly@chuv.ch (V.M.); tamila.abdurashidova@chuv.ch (T.A.); matthias.kirsch@chuv.ch (M.K.); 4 Department of Cardio-Respiratory Physiotherapy, Lausanne University Hospital (CHUV), 1011 Lausanne, Switzerland; caroline.botteau@chuv.ch (C.B.); marius.huber@chuv.ch (M.H.); 5 Faculty of Biology and Medicine, University of Lausanne (UNIL), 1011 Lausanne, Switzerland; jerome.graf@unil.ch

**Keywords:** cardiac anesthesia, enhanced recovery, perioperative medicine, outcome, opioid-sparing

## Abstract

**Background**: Enhanced Recovery After Surgery (ERAS) protocols have been implemented in various surgical specialties to improve patient outcomes and reduce opioid consumption. In cardiac surgery, the traditionally high-dose opioid use is associated with prolonged ventilation, intensive care unit (ICU) stays, and opioid-related adverse drug events (ORADEs). This study evaluates the impact of an ERAS^®^ Society-certified program on opioid consumption in patients undergoing elective cardiac surgery at Lausanne University Hospital. **Methods**: A retrospective, monocentric observational study was conducted comparing two patient cohorts: one treated with ERAS protocols (2023–2024) and a retrospective control group from 2019. Data were collected from the hospital’s electronic medical records and the ERAS program database. The primary outcome was total opioid consumption, measured intraoperatively and postoperatively (postoperative day (POD) 0–3). Secondary outcomes included pain control, length of stay, complications, and recovery parameters. Statistical analyses included multivariate logistic regression to identify factors associated with reduced opioid consumption. **Results**: Patients in the ERAS group demonstrated significantly lower total opioid consumption, whether intraoperatively (median sufentanil: 40 mcg vs. 51 mcg, *p* < 0.0001) or postoperatively (POD 0–3: *p* < 0.001). The ERAS group had faster extubation times, earlier mobilization and pain control with non-opioid analgesics, fewer complications, and shorter hospital stays (9 vs. 12 days, *p* < 0.001). Logistic regression identified fast-track extubation and absence of complications as strong predictors of reduced opioid use. **Conclusions**: The implementation of an ERAS protocol in cardiac surgery significantly reduces opioid consumption while enhancing recovery.

## 1. Introduction

Historically, anesthesia and analgesia protocols for cardiac surgery have relied heavily on high-dose opioids [1,2]. Despite advancements in anesthesia practices, significant variability in opioid dosing for cardiac surgery persists [3]. Over the past two decades, the cardiac surgery community has increasingly recognized that high-dose opioid use is associated with prolonged mechanical ventilation, extended intensive care unit (ICU) stays, and a range of adverse effects—including drowsiness, dizziness, delirium, respiratory depression, nausea, vomiting, and constipation. These complications can delay recovery by hindering mobilization and resumption of oral intake, ultimately increasing hospital length of stay and healthcare costs [4,5,6]. Additionally, prolonged opioid use increases the risk of tolerance and physical dependence.

Patients undergoing cardiac surgery face a considerable risk of long-term postoperative pain, with incidence rates as high as 37% within six months and persisting in 17% of cases two years after surgery [7]. Inadequate pain management often leads to increased opioid consumption [8], contributing to persistent opioid use, which affects up to 10% of patients six months post-surgery [9]. This risk is particularly pronounced among opioid-naive patients when daily doses exceed 50 morphine milligram equivalents (MME) or when large opioid quantities are prescribed at discharge [9,10].

Encouragingly, multimodal pain management strategies incorporating more than two non-opioid adjuvants have proven effective in reducing the likelihood of prolonged opioid use [10]. However, patients discharged on opioids face a 48% increased risk of hospital readmission within 30 days [11]. Moreover, the progression to opioid use disorder and overdose—occurring in up to 0.8% of patients within one year of surgery—underscores the importance of effective pain management and careful perioperative opioid use [12]. Recent expert opinions and consensus guidelines have emphasized opioid stewardship in cardiac surgery, aligning with current evidence and expert recommendations [13,14].

For over 20 years, the Enhanced Recovery After Surgery (ERAS^®^) Society has advocated for multimodal perioperative care pathways aiming to minimize postoperative complications, reduce hospital stays, and facilitate a quicker return to normal activities. A cornerstone of these pathways is optimizing pain management through multimodal protocols while reducing opioid use [15]. In 2019, ERAS recommendations were published for cardiac surgery [16]. Key components of these recommendations aim to address postoperative pain, reduce opioid-related adverse drug events (ORADEs), and limit overall opioid consumption.

Our cardiac surgery program at Lausanne University Hospital (CHUV) achieved ERAS^®^ Society certification in October 2023. As part of this certification, we have updated our pain management protocols to ensure the systematic use of multimodal analgesia. This study aims to evaluate the impact of ERAS protocols on opioid consumption after cardiac surgery.

## 2. Materials and Methods

The study was conducted in accordance with the Declaration of Helsinki, and the protocol was approved by the Ethics Committee of Commission cantonale d’éthique de la recherche sur l’être humain du Canton de Vaud (CER-VD) (CER-VD #2024-00632).

This was a retrospective monocentric observational study comparing two cohorts of patients undergoing elective cardiac surgery at Lausanne University Hospital: one cohort treated with cardiac ERAS protocols and one retrospective cohort from 2019. This study adheres to the STrengthening the Reporting of OBservational Studies in Epidemiology (STROBE) guidelines for reporting on observational studies. Data collection for the ERAS cohort was completed through the cardiac ERAS program database on Research Electronic Data Capture (REDCap) [17,18]. The data to be collected were predefined before the start of the program and patients were prospectively enrolled during the ERAS period. To minimize outcomes and reporting bias, the clinical outcomes measured were limited to those hypothesized to be affected by the cardiac ERAS protocol. Data collection for the retrospective cohort was carried out using the hospital’s electronic medical record system and integrated into the same REDCap database.

### 2.1. Participants

The cardiac ERAS program is an ongoing activity started at Lausanne University Hospital in May 2023. The last patient in the ERAS cohort was included in November 2024. The control group consists of patients who met the inclusion criteria during 2019, the last year in which the cardiac surgery program was not impacted by the COVID-19 pandemic.

Inclusion criteria were patients aged 18 years or older, undergoing elective cardiac surgery via median sternotomy with the use of cardiopulmonary bypass, regardless of surgical complexity or prior cardiac surgery history. Exclusion criteria included patients undergoing urgent surgeries, ventricular assist device implantation, or heart transplantation, or those who declined to provide general consent for data reuse.

### 2.2. ERAS Protocols

The certification process at our institution was recently published by Ltaief et al. [19]. To obtain the certification, the ERAS^®^ Society (Stockholm, Sweden) mandates a comprehensive system featuring well-defined, widely recognized protocols and adherence rates above 70%. These protocols integrate ERAS^®^ Cardiac Society guidelines by Engelman et al. [16], along with other enhanced recovery recommendations [20,21] and the service’s pre-existing perioperative management practices.

The patient’s perioperative journey is structured into seven key steps: the surgical consultation, where the surgical indication is established and the decision for surgery is made; the preoperative anesthesia consultation and ERAS nurse consultation; hospitalization before surgery; the day of surgery; the intensive care stay; the intermediate care stay; and the postoperative hospitalization. Eight general objectives were defined in our ERAS program and implemented transversally throughout the patient’s journey:

1. Education and empowerment of the patient.
2. Optimization of nutrition and glycemic control.
3. Optimal treatment of anemia and Patient Blood Management (PBM).
4. Infection prevention strategy.
5. Reduction of the duration of invasive ventilation and sedation.
6. Goal-directed hemodynamic therapy.
7. Optimization of pain management with a focus on reducing opioid use.
8. Early mobilization with timely removal of equipment.

The ERAS protocol for pain management involves multimodal analgesia during both the intraoperative and postoperative periods. In the intraoperative phase, dexmedetomidine is administered upon entry in the operating room at 0.5 mcg/kg over 10 min, followed by a continuous infusion of 0.5 mcg/kg/h until the end of cardiopulmonary bypass (CPB). Ketamine is administered simultaneously at 0.25 mg/kg over 10 min, followed by a continuous infusion of 0.25 mg/kg/h until the end of CPB. Magnesium sulfate is administered as a 4 g dose after intubation. Sufentanil is given as an initial dose of 10–15 mcg before intubation, with an additional 5 mcg dose if necessary during the operating period. Acetaminophen is administered as a 1 g dose at the time of sternum closure. Local infiltration or a parasternal intercostal plane block is used at the surgical site.

Postoperatively, acetaminophen is scheduled every 6 h, at doses of 500 mg to 1 g. Metamizole or nonsteroidal anti-inflammatory drugs (NSAIDs) are given every 8 h as needed in patients without contraindications. Opioids are reserved for patients with a Visual Analog Scale (VAS) pain score exceeding 4. If needed, intravenous (iv) morphine is administered as required as long as the patient has not resumed oral intake, then at a dose of 5–10 mg orally every 4 h as required, though opioid use is minimized. The opioid prescription is re-evaluated daily and adjusted based on the patient’s pain levels and needs. Opioids are discontinued once the chest tubes are removed, and tramadol may be considered only if the VAS pain score remains above 4 (Table 1).

Ondansetron is administered for postoperative nausea and vomiting (PONV) prophylaxis at a dose of 4 mg in the operating room, then daily if the patient requires opioids.

During the certification process, education was provided to caretakers involved in postoperative care of patients for pain assessment and adherence to protocols. Compliance with protocols was evaluated before certification and remains an ongoing process as part of the program.

### 2.3. Data Collection

Study data were collected and managed using REDCap electronic data capture tools hosted at CHUV [17,18]. The following baseline characteristics were collected: age, gender, body mass index (BMI), smoking status, alcohol consumption, recreational drug use, arterial hypertension, diabetes, dyslipidemia, chronic pulmonary disease, preoperative creatinine value, extracardiac arteriopathy, cerebrovascular disease, American Society of Anesthesiologists (ASA) physical status classification, and left ventricular ejection fraction (LVEF). The following characteristics of surgery were collected: type of surgery, operation, aortic cross-clamp and CPB duration, redo surgery, isolated cerebral perfusion, and systemic circulatory arrest.

### 2.4. Objectives and Outcomes

The primary objective was to evaluate the effectiveness of the cardiac ERAS program in reducing total opioid consumption. Secondary objectives included assessing the program’s impact on recovery-related and opioid-related outcomes, such as the duration of mechanical ventilation, mobilization, incidence of postoperative nausea and vomiting, return to bowel function, incidence of complications, and length of hospital stay. The primary outcome was total opioid consumption, which included intraoperative sufentanil (in micrograms) and all postoperative opioids administered from postoperative day (POD) 0 to 3, converted to morphine milligram equivalents (MME) using a standardized conversion method [22].

Secondary outcomes included the number of patients extubated within the first 6 h post-surgery (fast-track extubation) and the duration of mechanical ventilation in the ICU, maximum pain rating on the Visual Analog Scale (VAS) from POD 0 to 3, time to achieve pain control on oral analgesics (defined as a consistent VAS < 4), time to last opioid use (in days after surgery), incidence of mobilization at the first meal after extubation (defined as sitting in a chair for eating), mobilization on POD 1 to 3, administration of PONV prophylaxis, incidence of nausea and vomiting from POD 0 to 3, time to last drain removal (in days after surgery), time to bowel recovery (defined as time to first stool in days after surgery), number of patients discharged with tramadol, hospital and ICU length of stay (LOS), number of patients free from any complications, and incidence of complications including death, reoperation, acute confusional state, ICU neuropathy, pneumonia, lobar atelectasis, ileus or constipation.

### 2.5. Statistical Analysis

Continuous variables are expressed as medians and interquartile ranges (IQRs), while categorical data are presented as absolute numbers and percentages. The Chi-square test was used to analyze the association between categorical variables. Student’s *t*-test was applied to continuous variables following a normal distribution, whereas the Mann–Whitney U test was used for continuous variables that did not follow a normal distribution. Patients were compared before and after the implementation of the ERAS program in terms of their postoperative morphine consumption from postoperative day zero to day three. The association between higher opioid consumption and demographic variables, operative data, the occurrence of postoperative complications, and various actions related to the ERAS program (such as early extubation, early drain removal, etc.) was evaluated using univariate logistic regression, followed by multivariate logistic regression for significant relationships, both before and after the implementation of the ERAS program. The median value of the postoperative morphine milligram equivalent dose for the entire cohort was used to subdivide patients into high versus low opioid consumption in the postoperative period. Results of the multivariate regression analysis are expressed as odds ratios (ORs) with their corresponding 95% confidence intervals (CIs). For all the aforementioned analyses, reported *p*-values were two-tailed, with a value of less than 0.05 considered statistically significant. All statistical analyses were performed using Stata, version 18.0 (StataCorp LLC, College Station, TX, USA).

## 3. Results

Between May 2023 and November 2024, 248 patients were enrolled in the Lausanne University Hospital cardiac ERAS program. In 2019, a total of 376 planned cardiac surgeries were performed. For this study, 22 patients were excluded due to semi-urgent surgery, 11 due to ventricular assist device implantation, 11 due to transaortic valve implantation, 19 due to off-pump surgery, 24 due to consent refusal, and 127 due to lack of consent. The final 2019 control group consisted of 162 patients.

### 3.1. Baseline and Surgical Characteristics

The baseline characteristics of the patients are shown in Table 2. Patients in the ERAS group were younger and had a higher BMI. In contrast, more patients in the control group had an extracardiac arteriopathy and a history of cerebrovascular disease. The surgical characteristics are detailed in Table 3. The two groups were similar, with the exception that the ERAS group had a lower median duration of surgery, aortic cross-clamp time, and CPB time.

### 3.2. Outcomes

**Figure 1:**
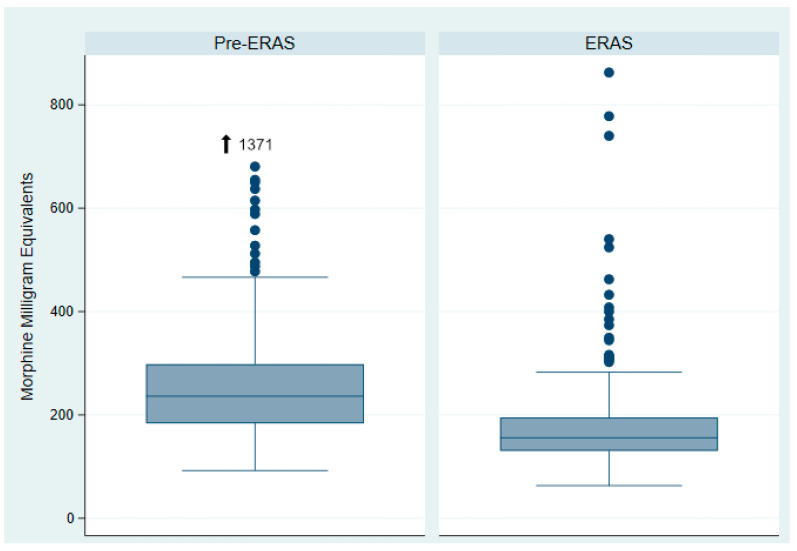
Total opioid consumption.

Since the implementation of ERAS in cardiac surgery, patients have demonstrated significantly lower total opioid consumption (Figure 1). Compared to the pre-ERAS cohort, patients in the ERAS group received significantly lower intraoperative doses of sufentanil (total sufentanil in mcg: 40 [30, 45] vs. 51 [40, 65], *p* < 0.0001; total sufentanil in mcg per kg: 0.48 [0.38, 0.58] vs. 0.69 [0.53, 0.87], *p* < 0.0001). From postoperative days 0 to 3, opioid consumption was also significantly reduced in the ERAS group compared to the pre-ERAS cohort (MME on POD0: 13 [6, 22] vs. 19 [8, 32], *p* = 0.0008; MME on POD1: 17 [10, 28] vs. 23 [14, 47], *p* = 0.0001; MME on POD2: 3 [0, 10] vs. 12 [5, 19], *p* < 0.0001; MME POD3: 0 [0, 0] vs. 5 [0, 12], *p* < 0.0001).

Secondary outcomes are summarized in Table 4 and Table 5. In the ERAS group, a greater proportion of patients were extubated in the operating room, and for those who were not, the duration of mechanical ventilation in the ICU was shorter. Despite a higher maximum VAS score on POD 0 to 3, patients in the ERAS group were more mobilized, achieved a VAS < 4 in less time, and consumed opioids for a shorter duration. Additionally, patients in the ERAS group had a shorter hospital length of stay and were more likely to remain free of complications.

**Figure 2:**
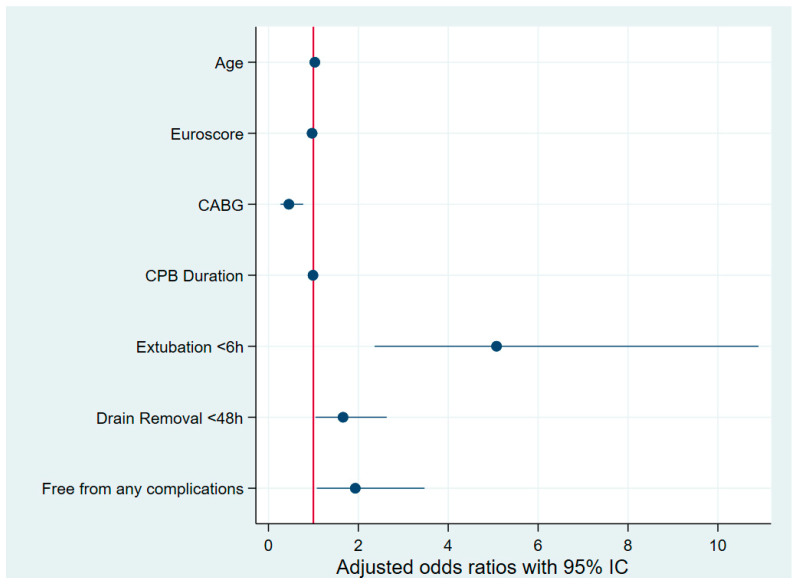
Forest plot of multivariate logistic regression for low postoperative morphine use in all the cohorts. CABG: coronary artery bypass grafting; CPB: cardiopulmonary bypass.

**Figure 3:**
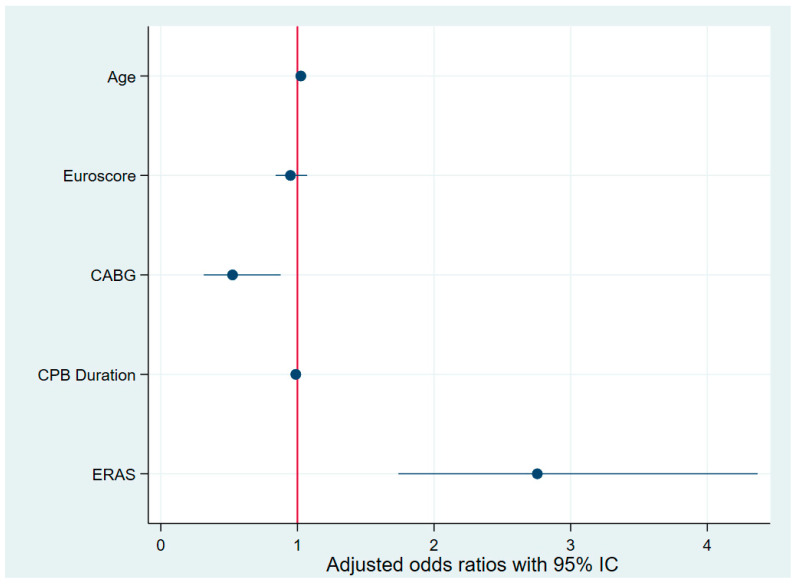
Forest plot of multivariate logistic regression for low postoperative morphine use in all the cohorts. CABG: coronary artery bypass grafting; CPB: cardiopulmonary bypass.

Multivariate logistic regression was applied to the control group to identify predictors of postoperative opioid consumption below the cohort median of 45 MME. After adjusting for age, EuroSCORE, and isolated CABG surgery, fast-track extubation was strongly associated with reduced opioid use (adjusted OR 12.015, 95% CI 2.661–54.253, *p* = 0.001). Additionally, the absence of complications was independently associated with reduced postoperative opioid use (adjusted OR for patients with any complications: 0.170, 95% CI 0.056–0.516, *p* = 0.002). A drain duration of less than 48 h showed a trend toward significance (adjusted OR 1.086, 95% CI 0.997–1.184, *p* = 0.059). Multivariate logistic regression was also applied to both groups. EuroSCORE was not associated with opioid consumption (adjusted OR 0.971, 95% CI 0.861–1.094, *p* = 0.625). CABG surgery (adjusted OR 0.455, 95% CI 0.268–0.773, *p* = 0.004) was associated with more opioid consumption. However, age (adjusted OR 1.032, 95% CI 1.012–1.0533, *p* = 0.002), CPB duration (adjusted OR 0.992, 95% CI 0.985–0.998, *p* = 0.012), fast-track extubation (adjusted OR 5.076, 95% CI 2.363–10.904, *p* < 0.0001), a drain duration of less than 48 h (adjusted OR 1.661, 95% CI 1.048–2.633, *p* = 0.031), and the absence of complications (adjusted OR for patients free from any complications: 1.933, 95% CI 1.075–3.475, *p* = 0.028) were independently associated with a consumption of less than 45 MME (Figure 2). Moreover, being included in the ERAS group (adjusted OR 2.756, 95% CI 1.739–4.369, *p* < 0.0001) was found to reduce opioid consumption (Figure 3).

## 4. Discussion

This study demonstrates that the implementation of an ERAS-certified protocol in cardiac surgery significantly reduces opioid consumption both intraoperatively and postoperatively. Our findings align with current literature advocating for Enhanced Recovery After Surgery programs, not only to improve postoperative recovery but also to minimize opioid-related adverse events.

Beyond all the measures in our ERAS program, the multimodal analgesia protocol systematically integrates dexmedetomidine, ketamine, magnesium sulfate, and acetaminophen as intraoperative adjuncts, combined with regional anesthesia techniques such as the parasternal intercostal plane block or surgical local anesthetic infiltration. Postoperatively, non-opioid adjuncts are the main cornerstone of pain management, with opioids only as rescue therapy and opioid prescriptions re-evaluated. This protocol aligns with current recommendations from the Enhanced Recovery After Surgery Cardiac Society and the PROSPECT Working Group of the European Society of Regional Anaesthesia and Pain Therapy [14,23]. This approach differs from our pre-ERAS practices, which relied heavily on long-acting opioids for postoperative analgesia. It is worth mentioning that dexmedetomidine, which was already used in 2019, was administered to 100% of patients without observed side effects, suggesting a good safety profile in an unselected cardiac surgical population.

Our results demonstrate that intraoperative sufentanil administration was significantly lower in the ERAS group. Compared to the commonly used intraoperative opioid doses in cardiac surgery, our sufentanil administration was lower [3] and can even be considered as low dose (≤2 mcg/kg of sufentanil or ≤50 morphine milligram intravenous equivalents) in current practices [24,25,26]. Since the use of a low dose has been demonstrated as safe practice [25,26], a further reduction toward what is considered an ultra-low dose (≤25 morphine milligram intravenous equivalents) may be achievable in the future [24]. This could be facilitated by a broader use of fascial plane blocks before surgical incision, the addition of NSAIDs, and more careful opioid titration, representing potential refinement to our intraoperative analgesia protocol moving forward.

A reduction in postoperative opioid consumption following the implementation of enhanced recovery protocols has been previously reported in the literature [27,28,29,30,31,32,33,34]. Interestingly, even before ERAS implementation, postoperative opioid consumption in our institution was already lower than the reported average in the literature, likely due to adherence to some ERAS principles prior to official program initiation, for instance, early extubation. Our findings reinforce the importance of a comprehensive system featuring well-defined protocols and high adherence rates, which were keys to our ERAS^®^ Society certification.

Predictors of reduced postoperative opioid consumption identified in our study include fast-track extubation, reduced drain duration, and absence of complications, as confirmed by logistic regression analysis. The safe use of early extubation has been previously described [26]. Notably, our pre-ERAS extubation times were already relatively low, and the ERAS protocol further optimized early extubation. One possible explanation for the reduction in opioid consumption with fast-track extubation is the improved pain assessment in non-sedated patients, which may allow for more accurate titration of analgesia. Intubated patients tend to receive continuous iv opioids as a common practice, potentially leading to higher overall consumption. Another key factor influencing opioid consumption is drain management. Delayed drain removal has been associated with increased opioid use. Our findings suggest that optimizing drain duration may further enhance recovery, constituting a potential area for improvement. However, one point on which we lack data is the number of drains the patients received. Therefore, we were not able to explore the possible relationship between the number of drains and postoperative pain and opioid consumption. Future protocols should focus on this topic.

Moreover, we found a strong association between participation in the ERAS program and reduced postoperative opioid consumption, with a multivariate logistic regression-adjusted odds ratio approaching 3. This improvement can be attributed to various ERAS components, including early extubation and early drain removal, and to a reduction in postoperative complications, all of which collectively contribute to faster recovery and minimized opioid requirements after surgery.

Concerning the influence of reduced CPB time, it is important to note that cardiac surgeons remained unchanged across both study periods and surgical techniques did not differ. However, optimizing surgical time, as with all the initiatives in ERAS programs, may contribute to a reduction in postoperative complications. Additionally, CABG surgeries are known to have extended pain locations compared to valve surgeries, related to internal mammary harvesting and area for radial artery or saphenous vein grafts [35]. This could explain the association between CABG surgery and increased postoperative opioid consumption. The lack of information in our database on the type of graft harvesting does not allow us to draw conclusions on postoperative pain related to harvesting sites. From the perspective of providing individualized pain management strategies to patients, this could be a future analysis to further enhance our protocols.

Postoperative pain control was achieved despite lower opioid use, although patients in the ERAS group reported higher maximum VAS scores. This discrepancy may be explained by earlier mobilization and increased physical activity in ERAS patients, leading to higher reported pain levels during movement. Moreover, patients reached a pain score below 4 more quickly, suggesting better pain stabilization with non-opioid analgesics. Before ERAS implementation, pain assessments were conducted in patients with minimal mobility, whereas ERAS encourages early ambulation, which may influence pain intensity and perception. This underscores the need for comprehensive pain assessments beyond static VAS scoring, incorporating functional recovery measures and patient-reported outcomes.

Postoperative complications in cardiac surgery, such as respiratory depression, excessive sedation, nausea, vomiting, and constipation, are influenced by multiple factors, including, amongst others, surgical duration, cross-clamp time, reinterventions, advanced age, preoperative stress, and anxiety. In this context, ERAS offers a comprehensive approach that considers these elements to optimize postoperative management. The earlier and more active mobilization demonstrates that patients were fit enough to take part in their recovery. Additionally, time to first stool was significantly shorter in the ERAS cohort, reinforcing the benefits of opioid minimization on gastrointestinal recovery. Apart from POD 2, the incidence of PONV was not reduced in ERAS patients, despite the daily administration of ondansetron if the patients used opioids. Enhancing compliance with intraoperative prophylaxis administration must be improved. Better risk evaluation of patients and the use of other molecules acting on different targets should be discussed in future protocol refinements.

Since ERAS implementation, tramadol use at discharge was significantly reduced to only 3.6% of patients. No other opioids were prescribed at discharge. This is a key achievement considering the well-established risks of prolonged opioid use and dependence following surgery. Moreover, our findings highlight the potential benefits of opioid stewardship programs in mitigating long-term use.

### 4.1. Limitations

Despite the strengths of our study, several limitations must be acknowledged. First, the retrospective and single-center nature of the analysis may introduce selection bias and potential inaccuracy in retrospectively collected data. However, the homogeneity of the populations and the rigorous data collection methodology limit these biases. Data collected were predefined before the implementation of ERAS protocols. As this strengthens the quality of data in the ERAS group, we were not able to rule out other possible confounding factors. Notably, our dataset lacks detailed information on postoperative non-opioid analgesic administration due to missing data in the 2019 cohort, limiting our ability to thoroughly analyze the impact of these medications on opioid reduction. Additionally, while we observed higher maximum VAS scores in the ERAS group, we could not assess patient satisfaction or average pain scores in a structured manner. Information on preoperative and postoperative chronic pain is also lacking. We are also missing data on persistent opioid use. Further prospective studies incorporating patient-reported outcomes and long-term follow-up will be essential to validate our findings.

Finally, the findings may not be directly generalizable to other institutions with different perioperative protocols, as the ERAS implementation process and institutional adherence rates, which are part of the certification we obtained from the ERAS^®^ Society, may have influenced outcomes.

### 4.2. Future Research Directions

Prospective randomized studies would be necessary to confirm these results and clarify the impact of the different components of the ERAS protocol on opioid reduction. Concerning multimodal analgesia, future directions for research should also explore additional regional anesthesia techniques, such as alternative nerve blocks, to further enhance opioid-free recovery. Further investigations on alternative opioid-sparing agents, including methadone, may also be conducted to improve pain management and recovery outcomes. Moreover, their use has already demonstrated significant value in this field [36,37,38]. The injection of bupivacaine into pleural and mediastinal drains is a simple approach that appears to be safe and efficacious in reducing postoperative pain related to drainage [39]. Opioid-free anesthesia is also an interesting strategy with early favorable results [40,41]. An ongoing randomized controlled trial will probably provide future arguments for these kinds of pain management strategies [42].

Standardizing patient-reported outcome measures will be critical to refining future ERAS protocols and ensuring patient-centered care. The integration of new strategies such as preoperative assessment of the risk of hyperalgesia or the individualization of analgesic protocols according to the patient profile could further improve these results. Finally, follow-up at three to six months after surgery could help identify patients with postoperative chronic pain and persistent opioid use, and to adapt our management strategies to prevent these issues.

## 5. Conclusions

The implementation of an ERAS program in cardiac surgery at CHUV has led to a significant reduction in opioid use. Our findings reinforce the importance of structured enhanced postoperative recovery protocols including opioid-sparing techniques, opioid stewardship, and patients’ empowerment in their recovery. Future prospective studies will be essential to confirm these results and further refine best practices in perioperative opioid stewardship.

## Figures and Tables

**Table 1:** ERAS multimodal analgesia protocol.

Intraoperative
Dexmedetomidine: 0.5 mcg/kg in 10 min, then 0.5 mcg/kg/h until end of CPB Ketamine: 0.25 mg/kg in 10 min, then 0.25 mg/kg/h until end of CPB Magnesium sulfate: 4 g after intubation Sufentanil: 10–15 mcg before intubation, 5 mcg repeated dose as needed Acetaminophen: 1 g at sternum closure Local infiltration or parasternal intercostal plane block
Postoperative
Acetaminophen: 500 mg–1 g scheduled every 6 h Metamizole or NSAID: PO every 8 h PRN
Opioids only if VAS > 4
Morphine: iv titration until patient has resumed oral intake, then 5–10 mg PO every 4 h PRN Daily re-evaluation of opioid prescriptions Discontinuation of opioids when chest tubes removed, then tramadol as a rescue if VAS > 4

CPB: cardiopulmonary bypass; NSAID: nonsteroidal anti-inflammatory drug; PO: per os; PRN: pro re nata; VAS: Visual Analog Scale.

**Table 2:** Demographic data.

Characteristics	Pre-ERAS (n = 162)	ERAS (n = 248)	*p* Value
Age, years, median [IQR]	67 [59, 74]	64 [55, 71]	0.010
Gender, n (%)			
Male	112 (69)	186 (75)	0.193
Female	50 (31)	62 (25)	
BMI, kg/m^2^, median [IQR]	25.75 [22.80, 29.63]	26.78 [23.91, 30.43]	0.018
Smoker, n (%)	40 (25)	63 (25)	0.921
Patients with alcohol overconsumption, n (%)	28 (17)	26 (10.5)	0.430
Patients with recreational drug use, n (%)	1 (0.6)	5 (2)	0.493
Arterial hypertension, n (%)	113 (70)	170 (69)	0.510
Diabetes, n (%)	28 (17)	56 (22)	0.194
Dyslipidemia, n (%)	78 (48)	144 (58)	0.061
Chronic pulmonary disease, n (%)	25 (15.4)	40 (16)	0.342
Extracardiac arteriopathy, n (%)	42 (26)	24 (10)	0.001
Cerebrovascular disease, n (%)	17 (10)	13 (5)	0.046
ASA class, n (%)	1: 0 (0) 2: 14 (8) 3: 121 (74) 4: 26 (16) 5: 0 (0)	1: 0 (0) 2: 31 (12) 3: 169 (68) 4: 46 (18) 5: 0 (0)	0.321
LVEF, %, median [IQR]	60 [55, 65]	60 [55, 65]	0.743
Preoperative creatinine value, mmol/L, median [IQR]	89 [73, 104]	85 [73, 97]	0.146

Data presented as median with interquartile range (IQR), or parameter counts (n) with percentage (%). BMI: body mass index; ASA: American Society of Anesthesiologists; LVEF: left ventricular ejection fraction.

**Table 3:** Surgery data.

Characteristics	Pre-ERAS (n = 162)	ERAS (n = 248)	*p* Value
Isolated CABG, n (%)	50 (30)	73 (29)	0.758
Other surgery, n (%)	112 (70)	175 (71)	0.758
Aortic cross-clamp duration, minutes, median [IQR]	72 [54, 98]	54 [42, 72]	0.001
CPB duration, minutes, median [IQR]	94 [71, 126]	73 [56, 91]	0.001
Operation duration, minutes, median [IQR]	227 [190, 274]	175 [144, 210]	0.001
Redo surgery, n (%)	24 (14)	19 (7)	0.021
Isolated cerebral perfusion, n (%)	6 (4)	5 (2)	0.425
Systemic circulatory arrest, n (%)	5 (3)	5 (2)	0.143

Data presented as median with interquartile range (IQR), or parameter counts (n) with percentage (%). CABG: coronary artery bypass grafting; CPB: cardiopulmonary bypass. Redo surgery is defined as patients who had already been operated on for cardiac surgery before.

**Table 4:** Intraoperative, ICU, and postoperative data.

Characteristics	Pre-ERAS (n = 162)	ERAS (n = 248)	*p* Value
Intraoperative PONV prophylaxis administration, n (%)	72 (45)	140 (59)	0.006
Fast-track extubation, <6 h, n (%)	77 (47.5)	185 (74.6)	0.0001
Duration of mechanical ventilation, hours, median [IQR]	2.15 [0, 5.7]	0 [0, 3.73]	0.0003
Drain removal, number of days since operation, median [IQR]	3 [2, 4]	3 [2, 4]	0.713
VAS, maximum value, median [IQR]	POD0: 6 [4, 7] POD1: 5 [4, 6] POD2: 4 [2, 6] POD3: 2 [0, 4]	POD0: 7 [5, 8] POD1: 7 [5, 8] POD2: 5 [3, 6] POD3: 3 [2, 5]	0.0013 <0.0001 0.0050 0.0163
Pain control on oral analgesics, VAS < 4, number of days since operation, median [IQR]	3 [1.5, 4]	3 [2, 5]	0.0087
Last opioid use, number of days since operation, median [IQR]	3 [2, 5]	2 [1, 3]	<0.0001
Patients discharged on tramadol, n (%)	21 (13)	9 (3.6)	<0.0001
Mobilization, at first meal after extubation, n (%)	4 (2.5)	118 (47.6)	0.0001
Mobilization, n (%)	POD1: 77 (47.5) POD2: 101 (62.3) POD3: 103 (63.6)	POD1: 185 (74.6) POD2: 196 (79) POD3: 181 (73)	0.0001 0.001 0.018
Observed nausea and vomiting, n (%)	POD0: 28 (17.3) POD1: 26 (16) POD2: 27 (16.7) POD3: 15 (9.3)	POD0: 33 (13.3) POD1: 38 (15.3) POD2: 22 (8.9) POD3: 26 (10.5)	0.319 0.125 0.004 0.822
Time to bowel recovery, number of days since operation, median [IQR]	4.5 [4, 5]	3 [3, 4]	0.0001

Data presented as median with interquartile range (IQR) or parameter counts (n) with percentage (%). ICU: intensive care unit; PONV: postoperative nausea and vomiting; VAS: Visual Analog Scale.

**Table 5:** Outcome variables.

Characteristics	Pre-ERAS (n = 162)	ERAS (n = 248)	*p* Value
Hospital LOS, days, median [IQR]	12 [9, 16]	9 [7, 12]	0.0001
ICU LOS, days, median [IQR]	1.08 [0.94, 2.12]	1.1 [0.94, 1.95]	0.563
Death, n (%)	3 (1.85)	0 (0)	0.031
Patients free of any complications, n (%)	20 (12.4)	60 (24.2)	0.003
Reoperation, n (%)	15 (9.3)	17 (6.9)	0.401
Pneumonia, n (%)	21 (13)	21 (8.5)	0.142
Lobar atelectasia, n (%)	10 (6.2)	18 (7.3)	0.670
Acute confusional state, n (%)	13 (8)	9 (3.6)	0.053
ICU neuropathy, n (%)	2 (1.2)	1 (0.4)	0.334
Ileus or constipation, n (%)	0 (0)	3 (1.2)	0.16

Data presented as median with interquartile range (IQR) or parameter counts (n) with percentage (%). LOS: length of stay; ICU: intensive care unit.

## Data Availability

The datasets used and/or analyzed during the current study are available from the corresponding author upon reasonable request.

## Abbreviations

The following abbreviations are used in this manuscript:

ERAS	Enhanced Recovery After Surgery
CHUV	Lausanne University Hospital
UNIL	University of Lausanne
ICU	Intensive care unit
ORADEs	Opioid-related adverse drug events
POD	Postoperative days
MME	Morphine milligram equivalents
CER-VD	Commission cantonale d’éthique de la recherche sur l’être humain du Canton de Vaud
STROBE	STrengthening the Reporting of OBservational Studies in Epidemiology
REDCap	Research Electronic Data Capture
PBM	Patient Blood Management
CPB	Cardiopulmonary bypass
NSAID	Nonsteroidal anti-inflammatory drug
VAS	Visual Analog Scale
IV	Intravenous
PO	Per os
PRN	Pro re nata
PONV	Postoperative nausea and vomiting
BMI	Body mass index
ASA	American Society of Anesthesiologists
LVEF	Left ventricular ejection fraction
LOS	Length of stay
IQR	Interquartile range
OR	Odds ratio
CI	Confidence interval
CABG	Coronary artery bypass grafting
DOAJ	Directory of open access journals
